# A school-based intervention programme to prevent anxiety and depression among Chinese children during the COVID-19 pandemic

**DOI:** 10.1186/s13034-024-00758-4

**Published:** 2024-06-17

**Authors:** Jiameng Li, Therese Hesketh

**Affiliations:** 1 Centre for Global Health, Zhejiang University School of Medicine, 866 Yuhangtang Road, Xihu District, Hangzhou, Zhejiang Province People’s Republic of China; 2 The Institute for Global Health, UCL, 30 Guilford St, WC1NEH London, UK

**Keywords:** Social emotional learning, Anxiety, Depression, Prevention, Chinese children

## Abstract

**Background:**

Child and adolescent mental health is a major public health concern worldwide. The development of children’s social and emotional skills helps to improve mental health and wellbeing, and prevent anxiety and depression. The school-based social emotional learning (SEL) programmes have proved effective in a number of countries. But in Mainland China, there has been no empirical research of the effectiveness on children’s mental health. The study conducted a SEL programme in China during the COVID-19 pandemic and aimed to determine whether: (1) a SEL programme can reduce anxiety and depression, (2) the intervention effect is influenced by sociodemographic characteristics, (3) the programme effects change children’s emotion management and communication.

**Methods:**

Participants were 230 children aged 8–12 years in the intervention school and 325 in the control school in two poor villages in central China. The study was a quasi-experimental trial, comprising 16 weekly 90-minute sessions. It used a mixed-methods design, with a quantitative survey administered at baseline, post-intervention, and 5-month follow-up, and qualitative interviews. Linear mixed effects regression modeling was used to analyse the intervention effectiveness, linear models were conducted to examine the moderation effect of sociodemographic variables, and the inductive thematic analysis approach was used for interview data.

**Results:**

The intervention had no significant effect on anxiety or depression, except that intervention school children who lived with neither parent (left behind children) reported lower depression scores than control school at post-intervention and 5-month follow-up. Qualitative interviews showed after intervention children were more able to control tempers and better communicated their thoughts and feelings, improving their relationships with family and friends.

**Conclusions:**

The programme was cheap, easy to implement, and warmly welcomed by children, schools and caregivers, suggesting it was feasible and potentially sustainable. More research is needed on the adaptation of the SEL programme in the Chinese context.

## Introduction

It is estimated that 10–20% of children and adolescents worldwide experience mental health problems  [4]. The internalizing disorders, depression and anxiety, are among the most prevalent mental health problems [30]. They display a marked comorbidity and share common components, such as maladaptive emotion regulation strategies, and a general tendency to experience a wide range of negative emotions [1]. Depression and anxiety in children are associated with adverse outcomes, such as poor school performance, relationship difficulties with peers, insomnia and the risk of suicide [31, 33]. Importantly, it is widely recognized that poor mental well-being in childhood or adolescence is a predictor of mental health disorders in adulthood [13].

Research into anxiety and depression in Chinese children and adolescents has started relatively recently. A study of 23,005 Chinese children and adolescents aged 8–18 years reported that the prevalence of depressive symptoms was 13% and anxiety, 22% [45]. A meta-analysis reported that the prevalence of depressive symptoms in Chinese children and adolescents aged 7–18 years was 19.9%, with higher prevalence in central and western China [31]. A group of children especially vulnerable to mental health problems are left-behind children (LBC). These are children left behind in rural areas by parents who migrate for work. Their numbers are currently estimated at 69 million [40]. LBCs are unevenly distributed in China, with the majority in central and western provinces, including Sichuan, Henan, Hunan, and Anhui, which export labour to eastern coastal provinces [10]. A recent meta-analysis reported that LBC were 1.7 times more likely to manifest depressive symptoms than non-LBC [41].

While the underlying determinants of mental health problems in these children cannot be easily addressed, specific measures can be taken to prevent and alleviate mental health problems. Developing children’s social and emotional skills is identified as a key factor in the improvement of children’s mental health and wellbeing, and supports them in achieving positive outcomes in school, work and life [19]. The concept and practice of social emotional learning (SEL) was first described in the US in 1994 (CASEL, 1994). The aim was to establish evidence-based SEL as an essential part of education from preschool through high school. SEL was defined as a process through which children acquire and apply the knowledge, attitudes, and skills necessary to understand and manage emotions, set and achieve goals, take the perspective of others, establish and keep positive relationships, and make responsible decisions [11]. SEL promotes the development of five interrelated cognitive, affective, and behavioral competencies considered to be important for success in school and life: self-awareness (e.g. recognizing emotions, strengths and limitations), self-management (e.g. regulating emotions and behaviors), social awareness (e.g. taking the perspective of and empathizing with others from diverse backgrounds and cultures), relationship skills (e.g. establishing and maintaining healthy relationships), and responsible decision making [42]. Several meta-analyses and reviews from a number of countries have reported that SEL programmes are effective in improving social emotional skills, as well as reducing or preventing mental health problems [9, 11, 35]. However, these studies have taken place predominantly in Western countries, especially the US [11], Australia [2, 3], and Europe [27, 29]. There are much fewer SEL programmes in non-Western settings, including a quasi-experimental study in Japan [23], implemented over eight weekly sessions among 63 six- to seven-year-olds, and the other in South Africa [26], which conducted across 10 weekly sessions among 46 twelve-year-olds.

In China the Ministry of Education initiated a pilot SEL programme in 2011 for children in five Chinese provinces in collaboration with UNICEF [39]. But there are no reports on either the process or the outcomes. There is no report on the use of a universal school-based SEL programme in the prevention of anxiety and depression among children in the under-developed rural region of China.

This programme was conducted to determine whether a school-based SEL programme was feasible in an under-developed area in central China during the COVID-19 pandemic, and specifically whether: (1) the programme can reduce symptoms of anxiety and depression, (2) the effectiveness of the programme is influenced by sociodemographic characteristics, (3) there was any change in children’s emotion management and ability to communicate thoughts and feelings with others. Consideration of the COVID-19 context was important to this programme. China adopted the world’s strictest control measures for COVID-19 from January 2020 to December 2022. This involved the implementation of diverse strategies to curb the spread of COVID-19 [7]. Over this three-year period, China conducted widespread testing and enacted rigorous contact tracing measures to identify and isolate individuals who had been in contact with confirmed cases. In the early stages of the COVID-19 pandemic in 2020, China implemented a series of strict control measures to contain the spread of the virus. These measures were particularly stringent in the first few months of the year, with public transportation suspended or restricted, schools, universities, and many businesses temporarily closed, and there was strict control of movement with many individuals forced to stay at home. On-line learning was introduced in many schools. From May 2020, as the situation improved and the number of cases decreased, China gradually eased lockdown measures and schools were not closed. Different regions in China experienced variations in the strictness of control measures based on the prevalence of the virus. From August 2021, some regions in China faced localized outbreaks, prompting swift and targeted responses to contain the virus [22]. The approach involved implementing strict measures in response to even small numbers of cases to prevent widespread transmission, followed by a gradual easing of restrictions when the situation improves. In December 2022, all COVID containment measures were lifted [43].

## Methods

The study was a quasi-experimental trial with a control group using a mixed-methods design [34].

### Participants

The programme was conducted in two primary schools (age range 7–12 years) located in poor villages of Henan province in central China. Around 40% of the children were left-behind. The two schools are 5 km apart and around 80% of the children were from households with incomes of less than 10,000 RMB (US$ 1369) per person in 2021, much lower than 19,000 RMB (US$ 2602), average for rural China [28]. In addition, fewer than 10% of the parents had attended high school.

All children across the six grades in the intervention school participated in the intervention, but first graders aged 7 years were excluded from the surveys, because of their difficulties in understanding the survey questions. In addition, six children aged 8–12 years in the 2nd -6th grades dropped out because they had some health issues or became close contacts during the COVID lockdown so that they were not able to continue their involvement. In the control school, the 2nd -6th grade children took the survey, four were excluded because they transferred to another school and five dropped out because of some health issues. In total, 555 were included in the final sample, 230 (54% female) from the intervention school and 325 (49% female) from the control school. The response rate of completion of all three assessments was 97.4% (555 of 570).

### Content of the programme

The SEL programme was administered over 16 weekly sessions throughout a semester from September 2021 to January 2022, during regular school hours, while COVID-19 restrictions were in place in China. Under the zero-COVID policy of that period, children were permitted to attend school because there was a very low incidence of COVID-19 in the area. Attending school was very important because in this poor rural area some children did not have access to on-line resources (e.g., stable WiFi) at home. Under the policy, students and teachers were required to adhere to a routine of daily temperature reporting and to undergo weekly nucleic acid tests to ensure ongoing monitoring and early detection of any potential cases. The programme facilitators were only allowed to attend the school once a week and were required to show nucleic acid tests before entering the school.

Each SEL session consisted of two 45-minute lessons, with a 10-minute class break after the first lesson. Throughout the programme, children were taught a variety of skills through a combination of activities, such as group discussion, role-play, story-telling, watching videos, handicrafts, and educational games. The programme was mostly adapted from the Ministry of Education-UNICEF social emotional learning resources, which were piloted in five Chinese provinces in 2011 [39]. The intervention sessions were developed and adapted on the basis of observation in the schools, fieldwork and interviews with 30 students and 6 teachers, to ensure that the specific needs of the local children were met. For example, to help children release anxiety and feel relaxed, comforting music were played in the warm-up games and children were led to practise meditation skills. Very few children spoke up in class, to ask or answer questions. To address this, we used videos of popular cartoons, in which characters demonstrate confidence, leading the children to discuss how to be confident. More than half of the interviewed children reported they had difficulties communicating with their parents, especially migrant parents, so we added several stories in the “expressing oneself” session to help with communicating with parents. We added a specific session “dealing with change” to help children of migrant workers. Several focus groups with children were conducted to identify which kind of games were most suited to the local context. Given the limited comprehension ability of younger children, discussion of stories and their meaning which was covered in higher grades were replaced with watching videos or playing educational games in Grade 2–3. For example, the role-play activity and story analysis of how one’s thoughts influence feelings and behaviours in Grade 4–6 was removed for lower grades. Instead, children in Grade 2–3 played the game rock-paper-scissors, and the winners and losers were invited to talk about their feelings. For example, the storytelling and discussions on collaboration in Grade 4–6 were replaced with a video showing Grade 2–3 students working together to find a treasure.

Overall the current programme differed from the Chinese Ministry of Education-UNICEF SEL programme in the following ways: the UNICEF SEL programme consisted of text only, while our programme included related videos in every lesson, such as cartoons of school bullying, and we included the relaxing music for meditation and lively music during warm-up. Because children enjoyed role-play, we added many role-play activities, such as how to say “No” and how to see things from others’ point of view. In the UNICEF SEL programme, there were sessions on story discussion, which the children found boring, so we replaced some of them with educational games, for example, making paper chains in groups to illustrate collaboration. The 16 sessions covered 5 topics, as shown in Table 1.

**Table 1:** Content of the intervention sessions

Topic	Session
1. Learning about emotions	1) Different and complex emotions
	2) How emotions influence behaviours
	3) Emotion management
2. Good to be me	1) Be confident and aware of strengths
	2) Expressing oneself and be oneself
	3) Be relax and calm down
3. Getting along with others	1) Showing kindness and care for others
	2) Understanding differences and resolve conflicts
	3) Communicating effectively and collaborate with others
	4) Dealing with change and loss
4. Saying NO to bullying	1) Bullying and being bullied
	2) Feelings of the victims
	3) What can we do if involved in bullying
	4) How to stop bullying
5. Moving toward your goals	1) Making a goal and a plan
	2) Strategies to overcome difficulties such as boredom and tiredness

### Procedure

The study was approved by the Zhejiang University Research Ethics Committee (protocol number ZGL202101-3). We contacted the local education bureau to explain the aims and contents of the programme, and asked for their support. They recommended five schools in the poorest villages of the local area and two schools were randomly selected. After obtaining permission from the schools, one was randomly assigned to the intervention and the other was assigned to the wait-list control. A pamphlet was given to the students and their caregivers with detailed information about the programme. Written informed consent was obtained from all participants and their caregivers in the intervention and control groups.

The SEL classes were led by a facilitator in each classroom. Six facilitators from the psychology department of a local university were recruited. They were trained over 7 three-hour sessions, which covered topics such as general theory and main content of the curriculum, how to conduct the intervention in the classroom setting, and introduction about the local setting. Facilitators were encouraged to think about daily examples in their experience that were relevant to the teaching of the sessions, and which would facilitate children’s understanding of the topics. They also had the chance to practise and role-play activities, and discussed how to optimise delivery of the sessions in the classrooms.

When the sessions were underway in the classrooms, one coordinator was responsible for three classrooms, observing and providing support if needed. The coordinator assessed the facilitators’ implementation, and gave feedback after each session to ensure the implementation fidelity of activities. After completing each session, the facilitators filled out a programme fidelity checklist, indicating which activities of each session were fully implemented, partially implemented or omitted, with explanation where appropriate. The facilitators’ checklist was compared with the results from the coordinator’s observations in order to determine the accuracy of facilitators’ reporting of programme fidelity. Since the programme was included in the school curriculum, children’s attendance rate was over 95% at each session.

Surveys, consisting of the same self-completion questionnaire for all participating children, were distributed at baseline one week before the intervention (pre-test, Assessment 1), one week after completion of the intervention (post-test, Assessment 2), and five months after the end of the intervention (follow-up, Assessment 3). Semi-structured interviews were conducted at Assessment 3. The informants were randomly selected from the class list of the intervention school. Interviews were carried out by the programme coordinator who was not directly engaged in the delivery of programme. Interviews were held one-to-one in a private room in the school. The duration of the interview ranged from 40 to 60 min. Interviews were audio-recorded and transcribed verbatim with verbal informed consent from the participants. Both paper questionnaires and interview audio recordings were anonymously coded according to students’ ID number and stored confidentially.

### Measurements

The draft questionnaire was piloted among 30 children from across the target age group. They were also asked to provide specific feedback about clarity, appropriateness, and any omissions. The final questionnaire had two parts:

(1) Sociodemographic and background information: gender, age, grade, number of siblings, the primary caregiver, whether living with parents, family economic status (retrieved from school records and then categorised into three levels-good, fair, poor), parents’ occupations, parents’ migration status, relationship with father and mother.

(2) The Revised Child Anxiety and Depression Scales (RCADS) was used to assess children’s anxiety and depression [8]. It is one of the most widely used instruments for the screening of anxiety and depression among 6 to 18 year olds [30]. The translation of the RCADS into Chinese was conducted following established guidelines for cross-cultural adaptation and linguistic validation. The translation process involved back-translation followed by a pilot testing phase to ensure comprehensibility and cultural appropriateness for the target population. Two of the six RCADS subscales were used in this survey- generalized anxiety disorder (GAD, 6 items) and major depressive disorder (MDD, 10 items). Each item is scored 0 to 3 corresponding to never, sometimes, often and always, with a higher score indicating poorer mental health. At baseline, Cronbach’s alpha was 0.83 for GAD, and 0.84 for MDD. At post-test, Cronbach’s alpha was 0.89 for GAD, and 0.86 for MDD. At follow-up, Cronbach’s alpha was 0.91 for GAD, and 0.89 for MDD.

The interview guideline included exploration of changes in children’s emotion management and communicating with others following the intervention, with the questions:

For children- (1) have you learnt anything about emotions? (2) do you know what emotion management is? (3) have you learnt ways to manage your emotions in the SEL classes? do you ever use them? (4) what about your communication with others before the SEL classes? (5) what about your communication with others after the SEL classes?

For caregivers or teachers- (1) do you think the children had problems managing their emotions and feelings before the SEL classes? (2) what about after the SEL classes? (3) did the children have good relationships with others before the SEL classes? (4) what about afterwards?

### Statistical analyses

First, baseline differences of the sociodemographic and background information between the intervention and the control group were compared through chi-square tests (for categorical variables) and independent sample t-tests (for continuous variables). Independent sample t-tests were also conducted to explore differences between the two groups of anxiety or depression at baseline. Second, linear mixed effects regression modeling (LMM) was conducted to analyze the effects of the intervention on anxiety or depression with observations nested within subjects. We specified random intercepts of subjects, and fixed effects of condition (intervention vs. control) and time (assessments). Third, to analyze age, gender and background information as possible moderators of the effects on anxiety or depression, we examined the interaction effects between condition (intervention vs. control) and the moderator through a series of linear models using changes in scores of anxiety or depression as the dependent variable. We examined short-term effects with changes from Assessment 2 to 1, and long-term effects with changes from Assessment 3 to 1. Data analyses were performed with R 4.2.1.

Interview data were analyzed using the inductive thematic analysis approach [6, 36]. The transcribed data were subjected to thematic analysis by the following steps: (1) generating initial codes, e.g. aware of anger, sadness; express thoughts, feelings; communicate more (2) sorting the codes into themes (3) gathering all relevant data to each theme (4) reviewing themes. The same strategy was applied for coding data obtained from children, school teachers and caregivers.

## Results

### Attrition

Fifteen children dropped out of the study, which is shown in Table 2. The dropping out rate did not differ by treatment group (χ^2^ = 0.01, *p* = 0.91). There was no significant difference in terms of sociodemographic and background variables at baseline between the retained sample (*n* = 555) and the dropout sample (*n* = 15). Dropping out wasn’t associated with the following factors at baseline: anxiety (GAD subscale) (t = − 1.12, *p* = 0.26) and depressive symptoms (MDD subscale) (t = − 0.68, *p* = 0.5).

### Sociodemographic and background information

The descriptive statistics for the sociodemographic and background information by group at baseline are presented in Table 2. At baseline, no difference was found between the intervention and control groups except that fewer children in the intervention group lived with both parents (*p* = 0.01), and fewer children in the intervention group reported they had good relationship with their mothers (*p* = 0.003).

**Table 2:** Sociodemographic and background information at baseline by treatment group. N (%)

	Total (555)	Control (325)	Intervention (230)	Attrition (15)
**Gender**				
Male	272 (49)	166 (51.1)	106 (46.1)	9 (60)
Female	283 (51)	159 (48.9)	124 (53.9)	6 (40)
**Grade**				
2nd	103 (18.6)	65 (20) *	38 (16.5)	3 (20)
3rd	116 (20.9)	64 (19.7)	52 (22.6)	3 (20)
4th	143 (25.8)	78 (24)	65 (28.3)	2 (13.3)
5th	109 (19.6)	57 (17.5)	52 (22.6)	6 (40)
6th	84 (15.1)	61 (18.8)	23 (10)	1 (6.7)
**The main caregiver**				
Grandparents	189 (34.1)	96 (29.6)	93 (40.4)	7 (46.7)
Father	34 (6.1)	23 (7.1)	11 (4.8)	2 (13.3)
Mother	327 (58.9)	202 (62.3)	125 (54.3)	4 (26.7)
**Live with**				
Both parents	326 (58.7)	201 (67.9) *	125 (55.8)	7 (46.7)
One parent	117 (21.1)	60 (20.3)	57 (25.4)	3 (20)
Neither parent	77 (13.9)	35 (11.8)	42 (18.8)	3 (20)
**Number of siblings**				
0	35 (6.3)	21 (6.5)	14 (6.1)	3 (20)
1	209 (37.7)	118 (36.3)	91 (39.6)	6 (40)
2	210 (37.8)	123 (37.8)	87 (37.8)	3 (20)
> =3	83 (15)	49 (15.1)	34 (14.7)	3 (20)
**Family economic status**				
Good	178 (32.1)	104 (34)	74 (33.6)	4 (26.7)
Average	245 (44.1)	136 (44.4)	109 (49.5)	4 (26.7)
Poor	39 (7.0)	28 (9.2)	11 (5)	1 (6.7)
Don’t know	64 (11.5)	38 (12.4)	26 (11.8)	4 (26.7)
**Relationship with father**				
Good	447 (80.5)	267 (82.7)	180 (78.6)	11 (73.3)
Average or poor	89 (16)	48 (14.9)	41 (17.9)	2 (13.3)
Do not know	16 (2.9)	8 (2.5)	8 (3.5)	2 (13.3)
**Relationship with mother**				
Good	478 (86.1)	289 (89.2) *	189 (82.5)	11 (73.3)
Average or poor	62 (11.2)	33 (10.2)	29 (12.7)	2 (13.3)
Do not know	13 (2.34)	2 (0.6)	11 (4.8)	1 (6.7)

*Note*: *p < 0.05; **p < 0.01; ***p < 0.001

### Change in anxiety and depression

#### The scores of anxiety and depression at three assessments

The descriptive statistics for anxiety and depression by treatment group at each assessment are presented in Table 3. At baseline there was no significant difference between the intervention and control group for anxiety, while the intervention group reported higher depression levels (t = − 2.33, *p* = 0.02).

**Table 3:** Descriptive statistics of anxiety and depression at three assessments by treatment group

		Control		Intervention		t
		*n*	Mean (SD)	*n*	Mean (SD)	
**Anxiety**						
	Assessment 1	306	5.39 (3.78)	210	5.5 (4.46)	− 0.34
	Assessment 2	309	5.64 (4.52)	209	6.0 (4.88)	− 0.84
	Assessment 3	309	5.92 (4.87)	210	6.22 (4.66)	− 0.71
**Depression**						
	Assessment 1	303	5.95 (4.48)	209	7.04 (5.67)	− 2.33*
	Assessment 2	309	6.05 (4.95)	209	7.16 (5.75)	− 2.29*
	Assessment 3	309	6.83 (5.96)	210	8.19 (5.69)	− 2.62**

*Note*: *p < 0.05; **p < 0.01; ***p < 0.001

#### Intervention effects on anxiety and depression

This part presents detailed results of two linear mixed models (LMMs) evaluating the intervention effects on anxiety and depression, with the pre− test depression scores controlled as a covariate. The report of the models in Table 4. followed the best practice guidance for LMMs (Meteyard and Davies [25]).

**Table 4:** Main and interaction effects in the linear mixed models of the intervention on anxiety and depression

Model on anxiety					
Fixed effect	Coefficient (B)	95%CI	SE	t	*p*
Intervention	− 0.39	[− 1.1, 0.3]	0.35	− 1.1	0.273
Assessment 2	0.24	[− 0.22, 0.71]	0.24	1.03	0.304
Assessment 3	0.51	[0.05, 0.98]	0.24	2.17	0.03
Intervention × Assessment 2	0.24	[− 0.49, 0.97]	0.37	0.65	0.516
Intervention × Assessment 3	0.17	[− 0.56, 0.89]	0.37	0.45	0.656
Random effect		Variance		S.D.	
Participant (Intercept)		6.94		2.63	
Residual		8.49		2.91	
Model fit					
R^2^		Marginal		Conditional	
		0.25		0.59	

Model on depression					
Fixed effect	Coefficient (B)	95%CI	SE	t	*p*
Intervention	0.27	[− 0.41, 0.95]	0.35	0.77	0.444
Assessment 2	0.12	[− 0.44, 0.67]	0.29	0.4	0.686
Assessment 3	0.85	[0.29, 1.41]	0.29	2.98	0.003
Intervention × Assessment 2	0.048	[− 0.83, 0.92]	0.45	0.11	0.915
Intervention × Assessment 3	0.29	[− 0.58, 1.17]	0.45	0.65	0.514
Random effect		Variance		S.D.	
Participant (Intercept)		2.55		1.6	
Residual		12.37		3.52	
Model fit					
R^2^		Marginal		Conditional	
		0.5		0.58	

*SE*-Standard error; *S.D*.- Standard deviation

There was no condition × time interaction effect (F = 0.22, *p* = 0.802) on anxiety, which suggested no difference between intervention and control groups of the changes in anxiety scores from Assessment 2 to 1, B = 0.24, 95% CI [− 0.49, 0.97], *p* = 0.516, or from Assessment 3 to 1, B = 0.17, 95% CI [− 0.56, 0.89], *p* = 0.656. There was no significant main effect of condition (intervention vs. control) (F = 0.8, *p* = 0.37). However, there was significant main effect of time (assessments) (F = 5.27, *p* = 0.005) on anxiety, which suggested children across the whole sample (both in intervention and control school) reported higher anxiety at Assessment 3, B = 0.51, 95% CI [0.05, 0.98], *p* = 0.03 than Assessment 1.

There was no condition × time interaction effect (F = 0.25, *p* = 0.78) on depression, which suggested no difference between intervention and control groups of the changes in depression scores from Assessment 2 to 1, B = 0.048, 95% CI [− 0.83, 0.92], *p* = 0. 915, or from Assessment 3 to 1, B = 0.29, 95% CI [− 0.58, 1.17], *p* = 0.514. There was no significant main effect of condition (intervention vs. control) (F = 2.65, *p* = 0.1) on depression. However, there was significant main effect of time (assessments) (F = 11.67, *p* < 0.001) on depression, which showed children of the whole sample reported higher depression at Assessment 3, B = 0.85, 95% CI [0.29, 1.41], *p* = 0.003 than Assessment 1.

To summarise, there was no intervention effect on anxiety or depression, and children both in intervention and control school reported higher anxiety and depression at Assessment 3 compared to Assessment 1.

#### Moderation analyses of the intervention effect on mental health

A series of linear models showed no interaction effect for short- or long- term effect on anxiety in terms of Condition × Gender, Condition × Age, Condition × Whether living with parents, Condition × Parents’ migration status, or Condition × other sociodemographic/background variables. However, a model showed “Condition × Whether living with parents” interaction short-term effect on depression, B = − 2.54, 95% CI [− 4.96, − 0.11], *p* = 0.04, which implied that children living with neither parent (compared with both parents) in the intervention school reported lower depression than the control school at immediate post-intervention compared to baseline. A model showed “Condition × Whether living with parents” interaction long-term effect on depression, B = − 2.96, 95% CI [− 5.76, − 0.15], *p* = 0.04, which implied that compared to children living with both parents, those living with neither parent in the intervention school reported lower depression than the control school at 5-month follow-up compared to baseline. A model showed “Condition × Relationship with father” interaction short-term effect on depression, B = − 2.53, 95% CI [− 4.73, − 0.34], *p* = 0.02, which implied that children having poorer relationships with fathers (compared to good) in the intervention school reported lower depression than the control school at immediate post-intervention compared to baseline.

### Implementation fidelity of the programme

There were overall 145 activities conducted in the programme. A summary of the number and percentage of activities fully implemented, partially implemented and not implemented is shown in Table 5, which compares programme fidelity assessed by programme facilitators and coordinators. According to the facilitators, the fully implemented rate was over 90% throughout the six grades, which was very similar to the coordinators’ assessment, except for the first grade. This indicated that the implementation fidelity was high, contributing to the internal validity of the study.

**Table 5:** Implementation fidelity assessed by facilitators and coordinators. N (%)

		Fully implemented	Partially implemented	Not implemented
First grade				
	Facilitators’ report	131 (90.3)	10 (6.9)	4 (2.8)
	Coordinator’s observation	125 (86)	15 (10.3)	5 (3.4)
Second grade				
	Facilitators’ report	131 (90.3)	11 (7.6)	3 (2.1)
	Coordinator’s observation	128 (88.2)	12 (8.3)	5 (3.4)
Third grade				
	Facilitators’ report	141 (97.2)	4 (2.8)	0 (0)
	Coordinator’s observation	140 (96.6)	5 (3.4)	0 (0)
Fourth grade				
	Facilitators’ report	135(93.1)	7 (4.8)	3 (2.1)
	Coordinator’s observation	132 (91)	9 (6.2)	4 (2.8)
Fifth grade				
	Facilitators’ report	136 (93.8)	8 (5.5)	1 (0.7)
	Coordinator’s observation	133 (91.7)	10 (6.9)	2 (1.4)
Sixth grade				
	Facilitators’ report	140 (96.6)	5 (3.4)	0 (0)
	Coordinator’s observation	138 (95.2)	7 (4.8)	0 (0)

### Qualitative interviews

All 8 school teachers from 2nd -6th grade in the intervention school were interviewed. The average age of the interviewed teachers was 38 years (range 26 to 52), seven of the school teachers had received college education and one high school education. Nine caregivers, five mothers and four grandmothers volunteered to participate in interviews. The average age of the caregivers was 44 years (range 35 to 57). Six caregivers had secondary school education, and three had primary school education. Six were homemakers and three worked part-time. We planned to randomly select 30% of children in the intervention school as interviewees, which is sufficient to reach data saturation. In the end we interviewed 36% of children because many children volunteered to be interviewed. Eighty-three children (36 boys and 47 girls) aged 8–12 years were interviewed, and 33 (40%) were left-behind children.

**Fig. 1:**
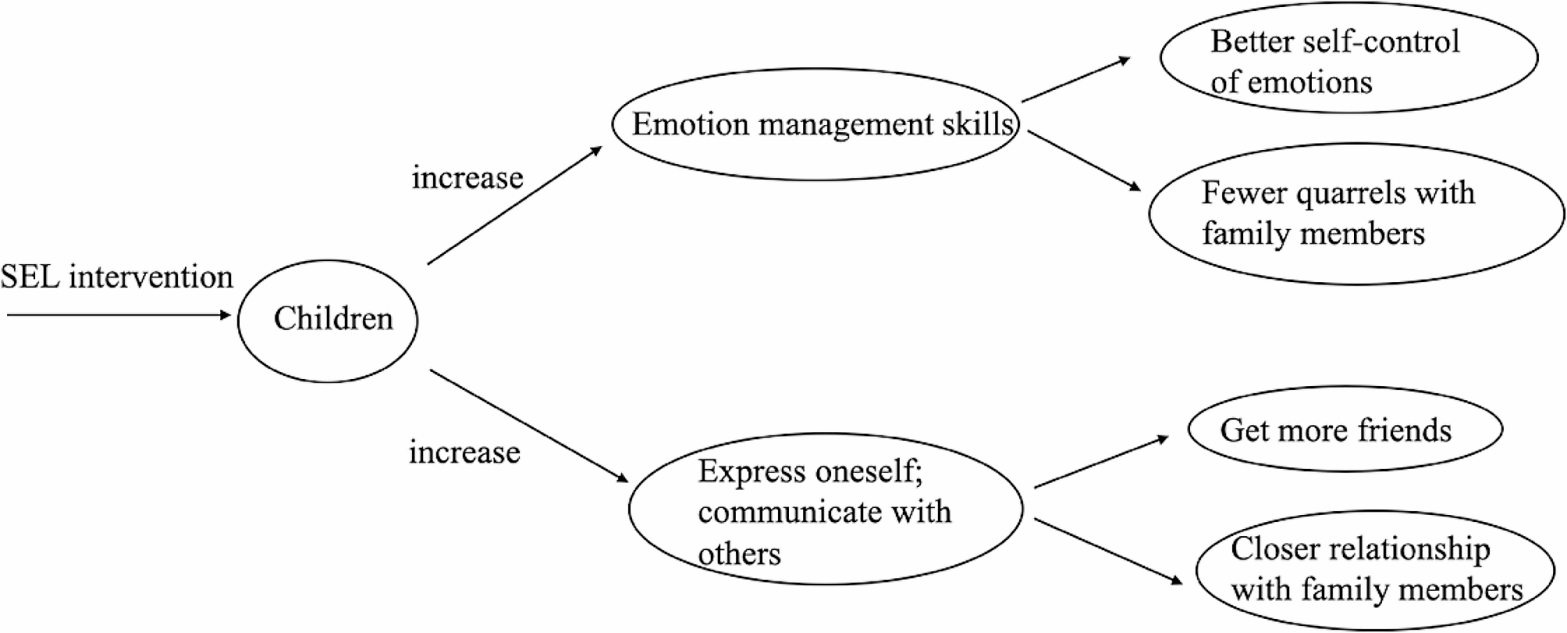
Conceptual framework of the SEL intervention on change of emotion management and communication

Two main themes were identified from the interviews. Following the SEL programme (1) Children could cope with negative emotions better. (2) Children were more likely to communicate thoughts and feelings. The results of the interviews informed a conceptual framework, which is shown in Fig. 1.

Theme 1 Children could cope with negative emotions better.

Over one quarter of the children (*n* = 23) reported they learned how to cope with negative emotions. The most commonly used coping mechanisms described were taking a walk, skipping, running, watching cartoons, and painting. A boy age 11, reported “*When I feel nervous now, I take a deep breath; when I am angry or sad, I think about something happy, or I do something I like.*” A girl age 10, said “*When I am unhappy, I write it down and then tear it up, which helps me forget.*” Three children learned to seek help from others. A boy age 12, stated “*Before*, *if I failed an exam and felt down, I didn’t know what to do, but now I talk to my parents, friends or go to a quiet place yelling out the pressure, which is really helpful.*” Meditation practiced in the SEL classes was also used by three children. A girl age 11, described “*When I feel sad, I listen to comforting music, close my eyes and imagine some peaceful images as the SEL teacher told me. This makes me forget the negative thoughts.*”

Two teachers reported that the SEL classes had helped children control their emotions. The 2nd grade teacher said “*One of the boys used to lose his temper and became aggressive with other children. Now after the SEL classes, when he gets angry, I ask him what the SEL teacher taught him. Now he takes a deep breath and calms down.*” The 3rd grade teacher reported “*After the SEL classes, children could control their emotions better, and are less likely to cry or shout.*”

Three caregivers reported that after the SEL classes children were less likely to lose their tempers. A mother of three children revealed “*My first daughter had a hot temper before and always lost control, crying and shouting over small issues. But she is much better now. She calms down and listens to me.*” Children learnt some skills to manage emotions. A mother of two daughters stated “*When my daughters feel angry, I ask them what the SEL teacher taught them. They take a deep breath, which makes them calm down and then they can solve the problem.*” Two caregivers reported that children dealt with negative feelings better than before. The grandmother of a boy age 10, reported “*I know that my grandson is often unhappy, but he is more positive after the SEL classes.*” Two caregivers reported children had fewer quarrels with them after the SEL classes. The grandmother of a girl age 10, said “*My granddaughter had a hot temper before, always lost control and we always had quarrels. However, nowadays she doesn’t shout when we have arguments. Now she is polite.*”

Theme 2 Children were more likely to communicate thoughts and feelings.

Nineteen children reported that since the SEL classes, they could express themselves better and show kindness to others. A girl age 10, said “*In the past, when my elder sister came home, I just sat there and didn’t say anything. But now I talk to her and show I am glad to see her.*” Four children reported that the SEL classes improved relationships with their families after they learned to communicate better. A boy age 9, stated “*Because of the SEL classes, I know how to comfort my mother when she is sad. So my mother talks to me more than before and we are getting closer. I am really happy about this.*” A boy age 10, told “*In the past, if I was feeling down, I kept it to myself and didn’t talk to others. But now I talk to my mother, and she listens carefully. This has made our relationship closer. My mother also thinks the SEL classes are very good for this.*” Five children commented that communicating more and not keeping things to oneself brings them more friends.

Four teachers revealed children were better able to express themselves after the SEL classes. A 5th grade teacher reported “*The SEL classes have made children happy and relaxed, and encouraged children to express themselves. They are now more likely to speak their thoughts and feelings. Some children never answered my questions in class and didn’t talk to others before, but they do now.*”

Two caregivers stated children started to talk more after the SEL classes. The mother of a girl age 12 reported “*My daughter really changed a lot in personality since the SEL classes. In the past, she was extremely introverted and didn’t tell me about what was going on unless I asked her, but now she tells me even if I don’t ask. She told me she wrote letters to the SEL teacher and always got reply, which helped her a lot.*” Two caregivers said they had closer relationships with their children after the SEL classes. The mother of a boy age 9 stated “When *we are together now, my son always has something to say to me. He never shared much about his life before the SEL classes. In the past, he only talked when I asked him. This has helped me to get to know him better. We are closer now.*”

In summary, following the SEL programme, children’s emotion management skills improved, thus they showed self-control and had fewer arguments with peers and family members. Children were also better able to communicate their thoughts and feelings, which may help to improve relationships. While only a limited number of children (just one quarter) expressed the changes.

## Discussion

The study was conducted to examine the intervention effects of a school-based SEL programme on anxiety and depression among primary school children in poor rural China amidst the COVID-19 pandemic. During that period, the strictness of COVID control measures varied in China based on the prevalence of the virus in different regions. There were very few COVID cases in this poor area in rural China, and local schools were not closed, so effects of COVID on the programme and children themselves were probably minimal. The key findings of the programme are: (1) there was no intervention effect on measures of anxiety or depression; (2) in the intervention school children who lived with neither parent (left behind children) reported lower depression than in the control school at post-intervention and 5-month follow-up; (3) after the intervention, children were able to better manage their emotions; (4) children were more likely to express themselves and communicate with others, which improved relationships with family members and friends.

This study overall failed to find intervention effects on the symptoms of anxiety and depression. There are a number of possible reasons for this. Anxiety and depression are partly linked to personality, so are a long-term characteristic especially in children, and therefore difficult to change, without targeted interventions. In addition, the lack of effect might be related to the programme itself: the intervention was short, and only a limited number of children had the chance to participate in the activities in the SEL classes because of large class size (García-Escalera et al. [14]). Facilitators led classes of around 50 children making it difficult to provide individual attention. Each session consisted of two 45-minute lessons, which were delivered on one afternoon each week. (The facilitators were allowed to attend the school only once a week due to COVID restrictions.) Some children, especially from lower grades, had difficulties focusing on the second lesson. The programme only targeted individual factors for depression and anxiety, but did not intervene with family or school-context factors, which are also associated with children’s internalizing symptoms (Roberts et al. [32]). Children both in the intervention and control group reported higher anxiety and depression at 5-month follow-up (June 2022) compared to baseline (September 2021). This increase over the school year may be due to an accumulation of difficulties together with the pressure of end-of-year examinations (Kozina [18]). Given the same measurement tools were used for the three assessments, children may have scored higher following the SEL classes, because of increased awareness of their problems. For children in the intervention group, the rising scores of anxiety and depression could also be explained by the sensitization effect (Ioannou [16]). Children in the intervention group had more emotional literacy and became more sensitive to their anxiety and depression symptoms as a result of the SEL intervention, causing them to recognize and report anxiety symptoms more readily than before the intervention. All these led to higher reported anxiety and depression levels during follow-up assessments.

Left-behind children, living with neither parent in the intervention school reported lower depression than the control school at post-intervention and 5-month follow-up compared to baseline, which suggested an intervention effect on depression in this sub-group. It’s been widely reported that left-behind children are at greater risk of depression compared to children living with parents (Liu et al. [21]). Parents’ departure and absence in children’s daily life is a predictor of depression among Chinese left-behind children (Sun et al. [37]). Lack of communication between left-behind children and their parents may lead to children’s alienation towards parents, and partly account for their increased risk of depression (He et al. [15]). The SEL programme included sessions which specifically supported left-behind children to deal with parental absence. Some of the interviewed children said they communicated more with others after the SEL classes. Positive interactions or close bonding with significant others such as caregivers or parents may decrease children’s vulnerability to depression (Liu [20]).

Although there was no intervention effect on anxiety or depression, qualitative results suggested most children learned how to better cope with negative emotions. The ability to modify negative emotions has been shown to predict lower levels of anxiety and depression (Berking and Wupperman [5]; Kassel et al. [17]). Depressed individuals have difficulties in identifying emotions, supporting themselves when experiencing negative emotions, and adaptively modifying emotions (Ehring et al. [12]). Individuals with anxiety disorders report poorer understanding of emotions, and less ability to recover after experiencing negative emotions (Mennin et al. [24]; Tull et al. [38]). The sessions on coping strategies for negative emotions delivered in our programme might protect children from developing anxiety or depression in future.

Given that no SEL interventions on children’s mental health have been evaluated in China, comparisons can be made with studies conducted in other East Asian countries. Two SEL programmes among primary school children in Japan reported conflicting outcomes- one found it was helpful for children’s resilience and mental health (Yamamoto et al. [44]), while the other did not improve children’s psychological well-being (Matsumoto et al. [23]). Our quantitative findings suggested there was no change in children’s anxiety and depression after the intervention. While the qualitative findings provided consistent evidence from children, teachers and caregivers that after the intervention children were better able to cope with negative emotions and communicate their thoughts and feelings, which might contribute to their mental health and wellbeing.

This study provides important perspectives about the implementation and feasibility of a universal school-based SEL programme in an under-developed rural region of China. It utilised a mixed-method approach and collected information from multiple sources (children, caregivers and school teachers), to get feedback about the implementation and effectiveness of the intervention. The intervention sessions were incorporated into the school curriculum, so children’s attendance was very high at each session, and the drop-out rate was low from baseline assessment to five-month follow-up.

However, this study has certain limitations. First, the self-report questionnaire measures might lead to social desirability bias. Second, the sample was small, with participants from only two schools, greatly limiting the generalizability of the findings. Third, one facilitator was responsible for 40–50 children in each classroom making it difficult to give enough attention and support to individual children, which might explain the lack of effectiveness. Fourth, at interview, selection of caregivers may have been biased, because they participated on a voluntary basis, and thus were more likely to be caregivers of children who benefited from the programme.

## Conclusion

This study has examined the effectiveness of the SEL programme on anxiety and depression among children in an under-developed rural region of China during the COVID-19 pandemic, but in an area where control measures were not strict and schools were not closed. It was not effective in reducing anxiety or depression, except among left-behind children. However, at interview, some children reported that they could deal with negative emotions better and were more likely to express their thoughts and feelings after this programme. The school-based programme was cheap, easy to implement by the non-professionals, and was warmly welcomed by children, schools and caregivers. It therefore has potential for feasibility and sustainability across different settings.

## Implications

More research is needed on the adaptation of the SEL programme to improve outcomes. There are some implications for future programmes: (1) facilitator-child ratio should be one-to-fifteen or less, (2) delivery of lessons twice per week on different days, (3) addition of a programme for families, including for migrant parents and caregivers. These may enhance the programme’s effectiveness in this setting.

## Data Availability

The datasets used during the current study are available from the corresponding author on reasonable request.
